# Impact of Community-Based Maternal Health Workers on Coverage of Essential Maternal Health Interventions among Internally Displaced Communities in Eastern Burma: The MOM Project

**DOI:** 10.1371/journal.pmed.1000317

**Published:** 2010-08-03

**Authors:** Luke C. Mullany, Thomas J. Lee, Lin Yone, Catherine I. Lee, Katherine C. Teela, Palae Paw, Eh Kalu Shwe Oo, Cynthia Maung, Heather Kuiper, Nicole F. Masenior, Chris Beyrer

**Affiliations:** 1 Center for Public Health and Human Rights, Bloomberg School of Public Health, Baltimore, Maryland, United States of America; 2 UCLA School of Medicine, Los Angeles, California, United States of America; 3 Global Health Access Program, Mae Sot, Thailand; 4 Burma Medical Association, Mae Sot, Tak, Thailand; 5 Karen Department of Health and Welfare, Mae Sot, Thailand; 6 Mae Tao Clinic, Mae Sot, Thailand; National Institute of Child Health and Human Development, United States of America

## Abstract

**Background:**

Access to essential maternal and reproductive health care is poor throughout Burma, but is particularly lacking among internally displaced communities in the eastern border regions. In such settings, innovative strategies for accessing vulnerable populations and delivering basic public health interventions are urgently needed.

**Methods:**

Four ethnic health organizations from the Shan, Mon, Karen, and Karenni regions collaborated on a pilot project between 2005 and 2008 to examine the feasibility of an innovative three-tiered network of community-based providers for delivery of maternal health interventions in the complex emergency setting of eastern Burma. Two-stage cluster-sampling surveys among ever-married women of reproductive age (15–45 y) conducted before and after program implementation enabled evaluation of changes in coverage of essential antenatal care interventions, attendance at birth by those trained to manage complications, postnatal care, and family planning services.

**Results:**

Among 2,889 and 2,442 women of reproductive age in 2006 and 2008, respectively, population characteristics (age, marital status, ethnic distribution, literacy) were similar. Compared to baseline, women whose most recent pregnancy occurred during the implementation period were substantially more likely to receive antenatal care (71.8% versus 39.3%, prevalence rate ratio [PRR] = 1.83 [95% confidence interval (CI) 1.64–2.04]) and specific interventions such as urine testing (42.4% versus 15.7%, PRR = 2.69 [95% CI 2.69–3.54]), malaria screening (55.9% versus 21.9%, PRR = 2.88 [95% CI 2.15–3.85]), and deworming (58.2% versus 4.1%, PRR = 14.18 [95% CI 10.76–18.71]. Postnatal care visits within 7 d doubled. Use of modern methods to avoid pregnancy increased from 23.9% to 45.0% (PRR = 1.88 [95% CI 1.63–2.17]), and unmet need for contraception was reduced from 61.7% to 40.5%, a relative reduction of 35% (95% CI 28%–40%). Attendance at birth by those trained to deliver elements of emergency obstetric care increased almost 10-fold, from 5.1% to 48.7% (PRR = 9.55 [95% CI 7.21–12.64]).

**Conclusions:**

Coverage of maternal health interventions and higher-level care at birth was substantially higher during the project period. The MOM Project's focus on task-shifting, capacity building, and empowerment at the community level might serve as a model approach for similarly constrained settings.

*Please see later in the article for the Editors' Summary*

## Abstract 2

Mullany and colleagues report outcomes from a project involving delivery of community-based maternal health services in eastern Burma, and report substantial increases in coverage of care.

## Editors' Summary

**Background:**

Every minute, somewhere in the world, a woman dies of complications related to pregnancy and childbirth. Access to essential maternal and reproductive health care (including family planning) is particularly bad in war-torn countries. In Burma, for example, where there have been decades of conflict between the military junta and ethnic minority resistance groups, the maternal mortality rate (the number of deaths among women from pregnancy-related causes per 100,000 live births) is around 380, whereas in neighboring Thailand it is only 44. Maternal health is even worse in the Shan, Mon, Karen, and Karenni regions of eastern Burma where ethnic conflicts and enforced village relocations have internally displaced more than half a million people. Here, the maternal mortality rate is around 1,200. In an effort to improve access to maternal health services in these regions, community-based organizations in Burma, the Johns Hopkins Center for Public Health and Human Rights, and the Global Health Access Program undertook an innovative pilot project—the Mobile Obstetric Medics (MOM) project—between 2005 and 2008. Local health workers from 12 communities in eastern Burma received training in antenatal care, obstetrics (the care of women during childbirth), postnatal care, and family planning at the Mae Tao Clinic in Mae Sot, Thailand. These “maternal health workers” then returned to Burma where they trained local health workers and traditional birth attendants to provide maternal health care to their communities.

**Why Was This Study Done?:**

Before the MOM project started, nearly 3,000 women living in the study communities were surveyed to evaluate the coverage of essential antenatal care interventions such as urine testing for infections during pregnancy, screening for malaria, and deworming; Urinary tract infections, malaria, and hookworm infections all increase the risk of poor maternal and neonatal outcomes. The preproject survey also evaluated how many births were attended by people able to deal with complications, and the provision of postnatal care and family planning services. In this study, the researchers undertake a similar postproject survey to evaluate the impact of MOM on the coverage of essential maternal health interventions among internally displaced communities in eastern Burma.

**What Did the Researchers Do and Find?:**

Between October 2008 and December 2008, trained survey workers asked nearly 2,500 ever-married women of reproductive age from the project's study communities about their access to antenatal and postnatal care, skilled birth attendants, and family planning. The results of the postproject survey were then compared with those of the “baseline,” preproject survey. The general characteristics (age, marital status, ethnicity, and literacy) of the women included in the two surveys were very similar. However, 71.8% of the women whose most recent pregnancy occurred during the implementation period of the MOM project had received antenatal care compared to only 39.3% of women surveyed at baseline. Similarly, among the women questioned during the postproject survey, 42.4% had had their urine tested and 55.9% had been screened for malaria during pregnancy compared to only 15.7% and 21.9%, respectively, of the women questioned in the preproject survey. Deworming had increased from 4.1% to 58.2% during the project, postnatal care visits within 7 days had doubled, and attendance at birth by people trained to deal with obstetric emergencies had increased 10-fold from 5.1% to 48.7%. Finally, the use of modern contraception methods (slow-release contraceptives, oral contraceptives, and condoms) had increased from 23.9% to 45.0%.

**What Do These Findings Mean?:**

These findings reveal a substantial improvement in access to maternal and reproductive health care in the study communities during the MOM project. However, because the study compared two independent groups of women before and after implementation of the MOM project rather than concurrently comparing groups of women who did and did not receive the services provided by the MOM project, this study does not prove that the MOM approach was the cause of the changes in the coverage of essential maternal health care. Nevertheless, these findings suggest that the type of approach used in the MOM project—the expansion of interventions (including components of emergency obstetric care) delivered outside healthcare facilities by community-based providers—might be an effective way to deliver maternal and reproductive health services in other parts of Burma and in other places where there are ongoing conflicts.

**Additional Information:**

Please access these Web sites via the online version of this summary at http://dx.doi.org/10.1371/journal.pmed.1000317.

- More information about the MOM project is available in previous publications by the researchers in PLoS Medicine, in Reproductive Health Matters, and in Social Science and Medicine
- Additional resources are also available on the MOM Project
- The Reproductive Health Response in Conflict Consortium provides information on how conflicts affect reproductive health
- The World Health Organization provides information on all aspects of health in Burma (in several languages)
- The Mae Tao clinic also provides general information about Burma and its health services
- The Burma Campaign UK and Human Rights Watch both provide detailed information about human rights violations, including those that affect maternal health in Burma
- The United Nations Population Fund provides information about safe motherhood and maternal and reproductive health during conflicts and among refugees (in several languages)

## Introduction

In settings of conflict, the additional reproductive and maternal health risks faced by women are well established [1],[2], and relief agencies increasingly recognize the importance of addressing them in crisis and conflict situations [3]. Collaborative groups including the Inter-Agency Working Group on Reproductive Health in Crises (IAWG) [4] and the Reproductive Health Response in Crisis Consortium (www.rhrc.org) have made substantial progress in areas of advocacy, tools to assist relief agencies in prioritizing and selecting essential services, and development of technical manuals and guidelines, and knowledge sharing through research and international conferences [5],[6]. However, few data exist to support the effectiveness of programs that specifically aim to increase coverage of essential maternal health interventions in settings affected by conflict. Relative to refugee settings, internally displaced communities have been particularly neglected [5], in part due to substantial difficulties in access by humanitarian agencies [3].

In eastern Burma, numerous temporarily or permanently displaced communities have been left vulnerable after decades of conflict between the State Peace and Development Council (SPDC) and ethnic minority resistance groups, and by resulting widespread human rights abuses [7]. Health status of communities in this region is poor, with child, infant, and maternal mortality rates far exceeding those estimated for the country as a whole [8]. This already precarious health status is worsened by well-documented and widespread human rights violations such as forced labor, forced relocation, destruction or theft of food supplies; exposure to these types of violations has been directly linked with increased risk of infant, child, and crude mortality rates and increased likelihood of landmine injuries, malnutrition, and malaria [9].

Between 2005 and 2008, community-based organizations in eastern Burma, representing Mon, Shan, Karenni, and Karen ethnic groups, collaborated with the Center for Public Health and Human Rights at Johns Hopkins University (www.jhsph.edu/humanrights) and the Global Health Access Program (www.ghap.org) to implement an innovative pilot project (“Mobile Obstetric Medics [MOM] Project”) to improve coverage of maternal health services among these vulnerable communities. A description of the project has been previously published [10] and is elaborated further below. The project emphasized bringing services directly to the target population via community-based providers rather than relying on a facilities-based approach where community members seek out services.

This project was evaluated in part through comparison of quantitative population-based surveys conducted at baseline prior to initiating service implementation (August–October 2006) and at the end of 2 y of field activities (October 2008–January 2009). Baseline data [11] demonstrated unacceptably low preprogram coverage of essential antenatal and postnatal interventions, extremely low access to skilled providers at birth (5.1%), and low use of modern contraceptives linked to high rates of unmet need (61.7%). In addition, recently delivered women who reported household exposures to human rights violations such as forced displacement or food security issues were at higher risk of anemia, had greater unmet need for contraception, and were substantially less likely to have preprogram access to core antenatal services [11]. In this manuscript we describe the impact of the MOM project on uptake of family planning, attendance at delivery by those capable of providing emergency obstetric care, and coverage of essential maternal health interventions.

## Methods

### Ethics Statement

The design and conduct of this study was reviewed and approved annually throughout the period of the project by the Institutional Review Board of the Johns Hopkins Bloomberg School of Public Health and the MOM Monitoring & Evaluation Committee, an independent committee located on the Thai/Burma border charged with overseeing monitoring efforts.

### Review of Design and Implementation of the MOM Project

The data for this manuscript were collected as part of the monitoring and evaluation component of the 2005–2008 MOM Project. The details of the design and implementation of this pilot project are provided in a prior publication [10]. In this section, the main elements of the project design and implementation strategy are briefly reviewed.

In 2005, leaders of the health committees of four ethnic groups of eastern Burma (Mon, Shan, Karen, and Karenni) collaborated with the Burma Medical Association, the Global Health Access Program, the Mae Tao Clinic in Mae Sot, Thailand, and the Center for Public Health and Human Rights at the Johns Hopkins University to design an innovative community-based strategy to improve access to essential maternal health interventions. While the long-term objective was to improve maternal and newborn survival this project was not designed to directly measure these broad outcomes. Rather, program effort and evaluation centered on the mechanisms through which such improvements might be realized. These mechanisms included improved access to focused antenatal care interventions, family planning, attendance at delivery by individuals trained to deliver one or more signal functions of emergency obstetric care, promotion of essential newborn care, and improved recognition of and care-seeking for maternal and newborn danger signs. The strategy rested upon establishing a three-tiered network of community-based providers: (1) traditional birth attendants (TBAs) provided improved antenatal care services, conducted normal deliveries, ensured that clean and hygienic practices were followed, and created links between community members and the upper-tiered workers; (2) Health workers (HWs) provided antenatal care and family planning supplies, attended deliveries, and provided universal misoprostol for prevention of postpartum hemorrhage and, when necessary, intramuscular antibiotics for sepsis. HWs also worked to strengthen links between TBAs and the highest tier workers; (3) Maternal health workers (MHWs) were responsible for overseeing the work of the TBAs and the HWs and, in addition to providing all of the interventions described above, attended as many deliveries as possible including both normal and complicated deliveries. This upper tier of workers was trained to provide components of basic and comprehensive emergency obstetric services, including community-based blood transfusion [10].

**Figure 1:**
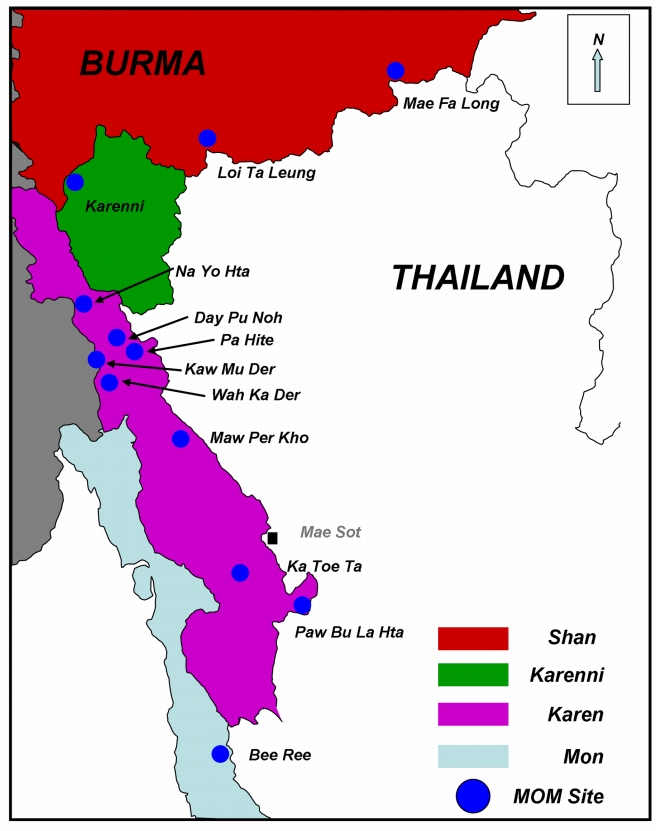
Map of Eastern Burma showing the MOM Project Communities. Map was previously published in Mullany LC, et al. [11].

The pilot project to implement this community-based three-tiered network of providers was conducted in a population of approximately 60,000 over four eastern Burma states in a total of 12 communities affected by conflict (Karen [8], Shan [2], Karenni [1], and Mon [1] States; see Figure 1). In mid-2005, an initial planning workshop, led by leaders of each ethnic health committee and attended by other local stakeholders and technical assistance partners, resulted in selection of the specific sites and identification of candidate individuals from those communities who could serve as MHWs. The first phase of the project was an 8-mo training phase for the MHWs (*n* = 33) at the Mae Tao Clinic in Mae Sot, Thailand. At this high volume clinic (approximately 2,000 deliveries per year), the main emphasis of the training was on practical skills building of the trainees in handling both normal and complicated deliveries. Specific obstetric skills included provision of intramuscular and intravenous antibiotics and MgSO_4_, manual removal of placenta, manual vacuum aspiration, administration of misoprostol for prevention and treatment of post partum hemorrhage, and assisted vaginal delivery (using portable Kiwi OmniCup). Workers were also trained in community-based blood transfusion, using a “walking blood bank” model. Under this model, prospective donors are pretyped and approached for blood donation at the time of an emergency, blood safety is assessed through a series of heat-stable rapid diagnostic tests, and direct person-to-person transfusion follows. Further details of this model have been described [10], and case studies of MHW's direct experiences with blood transfusion in the community have been summarized [12]. Throughout the training period, the trainees rotated through the clinic's labor/delivery ward and inpatient reproductive health while senior reproductive health workers and local and expatriate physicians provided direct supervision and tracked exposure of each trainee to the target procedures.

MHWs also built nonclinical skills including strategies for community mobilization and engagement, counseling, and training and supervision, as they were ultimately responsible for training the HWs and TBAs of their respective sites. The implementation phase of the project started in September of 2006 and focused on launching the newly established network to identify pregnant women and provide a range of antenatal and postnatal services, attend births, and deliver family planning services [10]. For the purposes of evaluation, this second phase closed on August 31, 2008. In practice, however, services continue to be provided by these networks under the leadership of the Burma Medical Association.

### Methods for Baseline and Endline Survey

The methods for the baseline (2006) and endline (2008) survey activities were largely identical and the implementation of the baseline survey was previously described [11].

### Training of Survey Workers

Individual survey workers (*n* = 16) were identified from each of the participating pilot communities and travelled to Mae Sot, Thailand to participate in a 3-wk training workshop. All workers were known to local community members and spoke the local language. Activities included (1) an initial orientation to the survey questions, definitions, and language-specific translations; (2) interviewing techniques (probing, establishing rapport, anchoring techniques); (3) practice through role-playing and simulated sampling exercises; and (4) detailed instruction on procedures (obtaining informed consent, rapid diagnostic tests, measuring malnutrition using mid-upper arm circumference (MUAC), and assessment of hemoglobin using a color scale [13]). These three measures were collected to assess malaria prevalence, malnutrition, and anemia as important indicators of the overall health status of women of reproductive age; the MOM Project interventions described above were not designed to impact these indicators at the population level. After training was complete, the surveyors traveled back to their respective communities inside Burma to conduct the surveys, visiting each selected cluster within their target area, and following the sampling procedure described below.

### Survey Instrument

Background and demographic variables such as age, education and literacy, and occupation were collected along with a brief pregnancy history. This information gathering was followed by a module focusing on access to antenatal care (ANC) during the current or most-recent pregnancy, and enabled tracking of coverage of malaria and anemia screening during pregnancy, iron/folate supplementation, antihelminthics, distribution of insecticide-treated nets, and overall number of antenatal visits and care providers. Questions on family planning and contraception enabled estimation of unmet need and use of individual methods. Respondents provided a household listing of members, and reported on any births or deaths that had occurred in the 12 mo prior to the survey. A final module contained questions on exposure to human rights violations such as food security issues, forced labor, and forced displacement; these variables have been associated with a range of health outcomes in eastern Burma [9],[11]. The survey instrument was translated into four languages (Burmese, Shan, Karen, and Mon) and was identical to that used in the initial baseline estimation of 2006 [11].

### Procedures

An oral script was followed to inform potential respondents of the purpose and procedures of the survey; data were only collected from those respondents providing consent to participate. After response to the question/answer part of the survey was complete, the respondent's MUAC was measured and women were screened for falciparum parasitemia using a rapid diagnostic test (Paracheck, Orchid Biomedical Systems). If positive, respondents who were not pregnant received a course of artemisinin combination therapy [14], while pregnant women were referred to the local MOM project worker. Hemoglobin level was estimated using a color scale (Teaching Aids at Low Cost) and those <8 g/dl were provided with 90 d of iron and folic acid and referred to a MOM project worker for follow-up. Women with levels 8–11 g/dl were referred to a MOM project worker for treatment and educational messages.

### Sampling Procedure

Sampling procedures were identical to that followed in the baseline period. To select survey respondents, two sampling schemes were followed to reach the desired sample size for each site; both schemes provide population-based representative samples within their respective areas. In Karenni (*n* = 1) and Karen (eight participating communities were grouped into four pairs based on geography) States a two-stage cluster-sampling scheme was followed. Within the one Karenni site and each of the four Karen subareas, 40 village-based clusters were selected proportionate to population size. Thus, the total intended sample in Karen State was 160 clusters of ten (1,600), and in Karenni State 40 clusters of ten (400). In each of the clusters in these regions, ten households with at least one ever-married woman of reproductive age was selected using proximity sampling. This second stage selection method was appropriate for these communities because household distribution in these village is not systematic [15],[16]. However, in the Shan (*n* = 1 area, two participating sites grouped together) and Mon (*n* = 1 area) States, where villages are few in number and households within villages are organized in rows, simple interval sampling of households was followed. In each case, the interval for systematic sampling interval was estimated as the total population divided by the number of required households (*n* = 400 in both Mon and Shan areas) [16]. Both of these methods result in samples that are representative within each area.

### Sample Size

Survey sample sizes for both baseline and endline were based on a balance of logistical constraints particular to working in this population and the desired level of precision of the estimates of progress in increasing coverage throughout the MOM project. The sample size calculation was based on the number of surveys needed to detect differences between the baseline and end-of-project estimates of the proportion of women receiving the key interventions such as antenatal care, family planning, and attendance at birth by those trained in providing components of emergency obstetric care (∼400 surveys/area). With four Karen areas and one area each from Mon, Shan, and Karenni State, the total planned sample size available for examining endline access to maternal health interventions was 2,800.

### Analysis

Surveys were returned to the MOM office in Mae Sot, Thailand, and entered into a secure password-protected database. Proportions, tabulations, and summary measures (mean, median, range, etc.) of coverage were estimated using binary, categorical, and continuous variables. Differences between samples from the two survey periods were assessed using chi-square tests and/or binomial regression models adjusted for clustering. Coverage for antenatal care services was estimated among those reporting a pregnancy within the period of the MOM pilot project. The proportion of women attended at birth by providers trained in emergency obstetric care was calculated, and coverage of postnatal care, early initiation of breastfeeding, and receipt of post partum vitamin A were estimated. Women with unmet need for limiting or spacing pregnancy were defined as follows: (1) nonpregnant women who were not using a modern contraceptive method to delay conception and who did not want any more children or wanted to delay conception beyond 2 y and (2) women who reported that they desired their current pregnancy to have been either avoided or delayed.

All these indicators above were compared between baseline and endline survey samples to estimate the impact of the pilot program. When comparing baseline and endline coverage for interventions received during the previous pregnancy, the 2-y MOM project period was compared to the 5-y period prior to baseline. The analysis was repeated for a 2-y time frame for baseline coverage, and no difference in program impact was observed. The 5-y comparison period was chosen as this was the time frame for previously published baseline estimates [11], and the slightly larger numbers provide improved precision for estimation of impact. Finally, changes in key coverage and intervention variables between baseline and endline were also analyzed separately for each of the four participating regions. Relative differences in coverage were modeled as ratios using binomial regression estimation with log link function. Variance estimates in the regression models were adjusted for the cluster-survey design using the “svy” suite within STATA 10.0 (StataCorp), the statistical package used for all analyses.

## Results

### Sampling Frame and Coverage

Data were collected between October and December 2008. The most up-to-date village-level population data (collected biannually monthly throughout the project) provided a sampling frame of 61,114 individuals, with an estimated 12,223 women of reproductive age (Table 1). Among 2,800 intended surveys across the four states, 2,484 (88.7%) potential participants were approached; among these 42 (1.7%) declined to participate, leaving 2,442 surveys for analysis. The main contributor to nonresponse was a loss of 22 clusters (i.e., 220 potential respondents) from Paw Bu La Hta in Karen State (Figure 1), where escalating conflict and attacks by SPDC troops during September and October of 2008 [17] prohibited access by the survey team workers.

**Table 1:** Survey sampling frame, coverage and response rate, and household size.

Sample Characteristic	Karen	Karenni	Shan	Mon	Overall
**Estimated population in pilot areas**	44,700	7,257	4,959	4,228	61,114
**Estimated reproductive-aged women (15–45 y)**	8,953	1,453	992	845	12,223
**Intended clusters**	160	40	N/A	N/A	200
Clusters reached	138 (86.3%)	37 (92.5%)	N/A	N/A	175 (87.5%)
**Intended surveys**	1,600	400	400	400	2,800
Surveys conducted	1,380 (86.3%)	367 (91.8%)	337 (100.0%)	400 (100.0%)	2,484 (88.7%)
Agreed to participate^a^	1,339 (97.0%)	367 (100.0%)	337 (100.0%)	399 (99.8%)	2,442 (98.3%)
Total sample within participating households	7,568	1,412	1,497	1,975	12,452
Mean household size (SD)	5.7 (2.1)	3.8 (1.9)	4.4 (1.7)	4.9 (1.9)	5.1 (2.1)

a Represents participation rate among those approached.

N/A, not available.

### Characteristics of Endline Sample and Comparison to Baseline

Characteristics of the respondents are shown in Table 2, along with similar information from the time of the baseline survey. The slightly lower endline proportion of Karen, relative to baseline, relates to the escalating conflict described above. The age of the respondents (mean 30.5 y), their educational (mean 2.6 y schooling) and literacy background (approximately half literate), marital status (95.4% married), and age at first marriage (mean 20.6 y) were similar to that of the sample interviewed prior to the MOM program in 2006. The proportion of respondents reporting ever being pregnant was slightly lower at endline (91.6%) than at baseline (95.1%); this difference is also reflected in higher mean age at first pregnancy (21.1 versus 20.8 y) and time since last pregnancy (2.6 versus 2.5 y), and slightly lower average number of pregnancies and live births, relative to preprogram numbers. Overall, respondents reported five neonatal deaths and 466 live births in the 12 mo prior to the survey resulting in a neonatal mortality rate of 10.7 (95% confidence interval [CI] 1.2–20.3). This number is approximately 54% lower than was estimated prior to the program (risk ratio [RR] = 0.46), but the population-based surveys were not designed to examine changes in mortality, and thus even this large difference in mortality was not statistically significant (95% CI 0.16–1.35).

**Table 2:** Comparison in demographic and socioeconomic characteristics between baseline (2006) and endline (2008) survey participants.

Indicator^a^	Baseline (*n* = 2,889)	Endline (*n* = 2,442)
Mean household size	5.4 (2.1)	5.1 (2.1)
Proportion Karen^b^	59.8%	54.5%
Proportion Buddhist	50.5%	47.5%
Age of respondent (y)	30.6 (7.5)	30.5 (7.5)
Educational level (y)	2.0 (2.8)	2.6 (3.2)
Literate (reading and writing)	52.5%	51.8%
Married	95.6%	95.4%
Age at marriage (y)	20.8 (5.0)	20.6 (4.0)
Ever pregnant	95.1%	91.7%
Age at first pregnancy (y)	20.8 (3.8)	21.1 (3.7)
Number of times pregnant	3.9 (2.6)	3.4 (2.4)
Number of live births	3.8 (2.3)	3.5 (2.1)
Time since last pregnancy (y)	2.5 (2.9)	2.6 (3.0)

a Continuous indicators are shown as mean (standard deviation), while binary variables are given in terms of percentage.

b The proportion Karen is a function of the sampling strategy and the response rate. In the 2008 endline survey, an entire Karen site was not accessed because of escalating conflict.

Health status indicators of respondents did not show any significant differences between baseline and endline samples. The distributions of hemoglobin were approximately equal (means at baseline and endline were 10.8 and 11.0 g/dl, respectively) and the proportion of all respondents with hemoglobin levels <11.0 g/dl was 61.1% in 2006 and 66.2% in 2008. As at baseline, respondents from families reporting theft or destruction of their food stocks or livestock (mostly from Karen State) were at higher odds of anemia than those not reporting such violations (OR = 4.28 [95% CI 2.84–6.44)]. Similarly, approximately one-fifth of respondents had MUAC measures <22.5 cm [18] in both samples, and positive rates for falciparum parasitemia were 7.4% and 8.2% in 2006 and 2008, respectively.

### Coverage of ANC Interventions

Among all ever-pregnant respondents, 1,531 (68.3%) women reported that their last pregnancy had occurred during the period of the MOM project and were included in the coverage analysis. Endline measures of access to antenatal care visits and critical services were substantially higher following implementation of the MOM pilot project (Table 3). The proportion of women who reported receiving at least one ANC visit during their last pregnancy nearly doubled, increasing from 39.3% to 71.8% (prevalence rate ratio [PRR] = 1.83 [95% CI 1.64–2.04]). Only about a third (34.4%) received the recommended number of visits (≥4), but this was also approximately doubled compared to baseline. Receipt of many key ANC interventions targeted by the MOM project exceeded 50%, and represented at least a doubling in coverage during the program period. The proportion of women receiving malaria testing during pregnancy (55.5%) and using an insecticide treated net (59.3%) was 2.53 (95% CI 2.01–3.18) and 2.75 (95% CI 2.19–3.45) times as high, respectively. Universal (presumptive) deworming with albendazole, recommended by the World Health Organization (WHO) for hookworm prevalent areas [19] but only rarely provided prior to the MOM project increased more than 14-fold from 4.1% to 58.2% (PRR = 14.18 [95% CI 10.76–18.71]).

**Table 3:** Changes in coverage of antenatal and postnatal interventions.

Service Provided	Baseline (*n* = 2,252)	Endline (*n* = 1,531)	PRR (95% CI)^a^
*Antenatal visit coverage*			
≥1 ANC visits	39.3%	71.8%	**1.83 (1.64–2.04)**
≥4 ANC visits	16.7%	34.4%	**2.06 (1.72–2.47)**
*Antenatal interventions*			
Blood pressure measured	43.1%	72.9%	**1.69 (1.51–1.89)**
Urine tested	15.7%	42.4%	**2.69 (2.05–3.54)**
Malaria test done	21.9%	55.5%	**2.53 (2.01–3.18)**
Positive rate	36.7%	11.8%	**0.32 (0.24–0.43)**
Tetanus toxoid			
≥1 dose	22.4%	15.6%	0.69 (0.47–1.03)
≥2 doses	14.3%	6.5%	0.46 (0.20–1.03)
90 d Fe/Folic Acid	11.8%	41.3%	**3.49 (2.80–4.35)**
Deworming treatment	4.1%	58.2%	**14.18 (10.76–18.71)**
Presumptive antimalarial provided	9.8%	12.5%	1.27 (0.93–1.75)
Used insecticide treated net	21.6%	59.3%	**2.75 (2.19–3.45)**
*Postnatal interventions*			
PNC visit within 7 d	33.7%	69.8%	**2.07 (1.81–2.37)**
Skin-to-skin care given	10.1%	27.2%	**2.70 (1.93–3.78)**
Maternal post partum Vitamin A	12.3%	63.4%	**5.17 (4.17–6.43)**
Breastfeeding initiated within 24 h	93.7%	95.8%	1.02 (0.99–1.05)

a Statistically significant comparisons are shown in bold.

Provision of other important ANC interventions also increased. Urine testing increased from 15.7% to 42.4% (PRR = 2.69 [95% CI 2.05–3.54]) and provision of 90 d of iron and folic acid supplements increased from 11.8% to 41.3% (PRR = 3.49 [95% CI 2.80–4.35]). Receipt of tetanus toxoid immunizations during pregnancy, which was not an intervention included in the MOM program package, was lower during the period of the MOM project, although the difference was not statistically significant. When these analyses were repeated with the baseline recall period restricted to 2 y, the estimates of program impact were similar (unpublished data).

### Labor, Delivery, and Postnatal Care

Data were recorded for all women reporting a pregnancy that ended in a birth (*n* = 1,305); this included live births that later died. Women largely delivered at home (82.9%), or at one of the mobile locations (12.0%) set up by the MHWs and/or the ethnic health committees in the target communities. Hospital delivery was rare (*n* = 55, 4.2%) and was predominately reported among Shan women who, due to border proximity and low levels of active conflict, could access Thai clinics or hospitals (*n* = 45). While endline place of birth did not vary substantially from baseline, the skill level of providers present during delivery increased significantly. At baseline, only 5.1% of women who had been pregnant during the previous 5 y reported being assisted by someone trained to provide emergency obstetric care. During implementation of the program, that figure increased nearly 10-fold. Among the 1,303 women reporting the delivery attendant for pregnancies ending during the MOM project period, 634 (48.7%) reported that a doctor (5.0%) or an MHW (44.4%) attended the delivery (PRR = 9.55 [95% CI 7.21–12.64]). More than one reported delivery assistant was possible; other providers mentioned were TBAs at 64.1%, which was comparable to baseline (64.6%) and relatives/friends at 22.0%, which was lower than at baseline (37.1%).

Postnatal care interventions targeted by the MOM project increased relative to baseline (Table 3, lower panel). Any visit to the new mother and baby within 7 d of delivery increased from 33.7% to 69.8% (PRR = 2.07 [95% CI 1.81–2.37]). Similarly strong relative increases in skin-to-skin contact and provision of postpartum vitamin A were observed. The proportion of mothers initiating breastfeeding within 24 h of birth was high prior to (93.7%) and during (95.8%) the MOM project period.

### Family Planning

Desire for more children did not change; approximately 40% of all women responding to this question in both survey periods did not want more children (Table 4). In contrast, greater availability of free modern methods of contraception during the MOM project period was associated with increases in effective contraceptive practice. The proportion of women reporting an action to delay pregnancy was higher in the 2008 sample (46.7%) than in 2006 (25.3%) (PRR = 1.84 [95% CI 1.61–2.12]). Depo-provera remained the most popular method, increasing in absolute utilization from 18.6% to 28.7%, while oral contraceptives more than tripled from 5.2% to 16.3% (PRR = 3.13 [95% CI 2.51–3.92]). While overall condom use remained low, there was a large relative increase from 1.4% to 6.3% (PRR = 4.51 [95% CI 2.59–7.87]). As a result of these increases in the reported use of modern methods, the unmet need for contraception in this population decreased from 61.7% in 2006 to 40.5% in 2008, a relative reduction of 35% (95% CI 28%–40%).

**Table 4:** Impact of program on family planning use and unmet need.

Indicator/Method	Baseline		Endline		PRR (95% CI)^c^
	*n*	Percent	*n*	Percent	
Do not want more children^a^	1,136	42.0	950	41.6	0.99 (0.91–1.07)
Doing anything to delay pregnancy^b^	725	25.3	1,110	46.7	**1.84 (1.61–2.12)**
Using a modern method^b^	685	23.9	1,070	45.0	**1.88 (1.63–2.17)**
Oral contraceptives	149	5.2	388	16.3	**3.13 (2.51–3.92)**
Depo-provera	531	18.6	682	28.7	**1.54 (1.34–1.78)**
Intra-uterine device	6	0.2	30	1.3	**6.01 (2.31–15.7)**
Norplant	4	0.1	1	0.0	0.30 (0.31–2.90)
Condoms	40	1.4	150	6.3	**4.51 (2.59–7.87)**
Sterilization	25	0.9	17	0.7	0.82 (0.42–1.59)
Unmet Need for Contraception^2^	1,764	61.7	963	40.5	**0.65 (0.60–0.72)**

a Baseline, *n* = 2,702; Endline, *n* = 2,283.

b Baseline, *n* = 2,861; Endline, *n* = 2,377.

c Statistically significant comparisons are shown in bold.

Analysis of program impact was repeated separately for each of the four states (Table 5). There was a trend toward larger relative increases in the Karen and Karenni regions in antenatal and postnatal intervention coverage, attendance at delivery by those trained to handle obstetric emergencies, and use of modern methods of contraception, compared with the Shan and Mon regions.

**Table 5:** Comparison of changes in key outcomes by region.

Outcomes	Karen		Karenni		Shan		Mon	
	Percent^a^	PRR^b^ (95% CI)	Percent^a^	PRR^b^ (95% CI)	Percent^a^	PRR^b^ (95% CI)	Percent^a^	PRR^b^ (95% CI)
*Antenatal/postnatal care*								
≥4 ANC visits	17.3	2.38 (1.50–1.75)	17.3	3.33 (1.38–8.02)	82.4	1.86 (1.43–2.31)	81.2	1.68 (1.32–2.14)
Blood pressure measured	66.7	2.09 (1.79–2.44)	85.6	1.63 (1.37–1.93)	83.7	1.34 (1.05–1.71)	80.9	1.18 (0.97–1.44)
Urine tested	34.3	4.56 (3.24–6.42)	70.7	5.93 (3.81–9.22)	78.7	2.47 (1.68–3.63)	27.4	0.62 (0.37–1.04)
Malaria test done	48.5	3.15 (2.48–4.01)	79.1	3.35 (2.38–4.70)	77.9	3.78 (2.29–6.26)	48.8	0.95 (0.51–1.79)
90 d Fe/Folic Acid	27.8	4.55 (3.04–6.80)	68.3	14.8 (5.67–38.7)	55.6	1.24 (0.63–2.44)	68.0	3.47 (1.83–6.57)
Deworming treatment	55.1	50.5 (30.2–84.6)	67.3	7.86 (4.09–15.1)	64.3	8.36 (5.34–13.1)	58.9	5.79 (3.92–8.54)
Used insecticide treated net	45.7	2.76 (2.11–3.60)	76.9	5.24 (3.52–7.80)	72.1	2.30 (0.49–10.7)	93.9	2.12 (1.73–2.63)
PNC visit within 7 d	63.8	2.33 (1.89–2.87)	77.8	3.26 (2.46–4.31)	74.6	1.18 (1.03–1.35)	91.1	1.85 (1.43–2.39)
EmNOC-trained attendant	41.8	22.4 (14.5–34.5)	85.2	16.7 (9.84–28.4)	76.2	5.59 (3.23–9.67)	44.5	3.39 (1.84–6.27)
*Family planning*								
Delaying pregnancy	33.9	3.73 (2.81–4.95)	38.9	2.60 (1.74–3.89)	68.1	1.10 (0.85–1.42)	74.2	0.91 (0.80–1.03)
Using a modern method	33.6	3.98 (2.99–5.31)	36.1	2.56 (1.67–3.93)	61.7	1.11 (0.94–1.31)	74.2	0.91 (0.79–1.05)
Oral contraceptives	16.9	5.05 (3.33–7.66)	13.0	1.70 (0.82–3.50)	8.5	0.88 (0.14–5.62)	17.8	2.22 (1.15–4.26)
Depo-provera	14.8	3.64 (2.46–5.41)	19.7	1.90 (1.13–3.18)	49.7	1.15 (0.89–1.48)	65.7	0.87 (0.78–0.98)
Condoms	8.9	24.4 (8.81–67.7)	8.2	2.06 (0.83–5.12)	6.4	2.64 (1.32–5.27)	0.0	N/A
Unmet Need	50.8	0.67 (0.60–0.75)	38.5	0.51 (0.42–0.62)	23.4	0.66 (0.39–1.11)	21.6	1.29 (0.81–2.05)

a Perecentage reported at endline survey.

b Relative change from baseline.

N/A, not available.

## Discussion

The community-based maternal health services delivery strategy promoted in this pilot project was associated with substantial increases in access to a range of essential maternal health services. In particular, the three-tiered network of providers led to an almost 10-fold increase in the proportion of women attended to at delivery by individuals trained to provide emergency obstetric care. Further, coverage and comprehensiveness of ANC increased substantially, with large absolute and relative increases in screening for hypertensive disorders of pregnancy, urine testing, malaria, and provision of deworming, iron folate, and insecticide-treated nets. Similar increases were seen for postnatal visits within 7 d and targeted postnatal interventions such as post partum vitamin A distribution and promotion of skin-to-skin contact. This tiered network of providers was also able to meet the need for family planning services that was clearly expressed by the community at the outset: unmet need was decreased by 35% largely owing to increases in the use of oral contraceptives and depo-provera, and smaller absolute increases in condom use. All these improvements were observed after implementation of an innovative community-based approach to service delivery in the context of ongoing conflict, security concerns, and human rights violations.

There are few comparable data from other community-based programs aiming to improve coverage of maternal or reproductive health interventions among internally displaced persons, especially in complex emergencies. In general, even after the acute phase of humanitarian crises, the specific health needs of women, or reproductive health needs more broadly often remain neglected, yet the need and the demand for such services is high [5]. Recognition of the international community's failure to meet these needs has largely spurred the advances in advocacy for comprehensive and coordinated responses to reproductive health concerns in conflict settings [20]. While there has been some progress toward improved epidemiological data, the need to translate information into programs and demonstrate success in program implementation with high coverage remains a substantial challenge. Furthermore, in the limited settings where change in coverage of services in refugee, internal displacement, or other conflict settings has been documented, services have often not been sufficiently comprehensive [21]. For example focus has more often been limited to increasing the number of ANC visits, without concurrent efforts to expand the proportion of women assisted by attendants with the knowledge and skills to handle complicated deliveries. The MOM project focused on more than quantity of visits by prioritizing the provision of individual evidence-based antenatal, delivery, and postnatal care interventions considered most critical to improving health outcomes. Furthermore, a concurrent effort to expand access to family planning supplies led to striking improvements in utilization and decreases in unmet need, thereby reducing women's exposure to the risks of pregnancy (planned and unplanned).

This pilot project evaluation was limited by a number of factors. Direct external oversight of surveyors during data collection was not possible given the unstable security status of the target communities. However, the training period prior to collection was extensive, time between training and data collection short, and survey instruments were identical to those previous used, and the overall implementation approach reflected refinement and lessons learned through 8 y of conducting population-based cluster-sampling surveys in these populations [8],[9]. While the sampling scheme is population-based and, within each specific area, is representative of the population in the pilot program areas, both the baseline and endline results here are not necessarily representative of the broader population in Karen, Karenni, Mon, and Shan States. Generalizing results beyond the program area is especially cautioned for Karenni, Mon, and Shan States where the program was conducted within a single target area. Further work with broader participation from these three regions as well as other underserved regions in Burma would allow for improved characterization of the maternal health needs in those settings and the degree to which strategies based on the MOM pilot project model might address those needs.

Loss of data from an entire site was an unfortunate reality of working in this setting. While there is no direct way to quantify the loss of these 22 clusters from one of the Karen areas (Paw Bu La Hta), it is possible that overall program impact might be underestimated because (1) there was a trend toward larger relative improvement in Karen communities, and (2) clusters were lost from communities with the lowest numbers at baseline and thus the largest scope for absolute improvements. As the program was a pilot project to examine the feasibility and effectiveness of a comprehensive community-based delivery strategy, activities were focused among a relatively modest number of sites and total population (∼60,000), with a security and access profile that was likely better than some of the most unstable regions of eastern Burma. The term “better” is clearly relative to this context; the program operated in a setting with substantial security concerns. One of the participating communities in northern Karen State was attacked by the SPDC in 2006 and the entire population was forced to move en masse into an adjacent area. MHWs participating in focus group discussions [12] reported numerous ongoing security-related challenges that prevented them from reaching a higher number of deliveries.

The main goal of the project was to demonstrate that proven maternal health interventions could be delivered in this unstable setting. Although we would expect that improved delivery of interventions such as deworming, malaria screening, and family planning would improve health outcomes, this may not have been the case at the population level. Data from the baseline survey [11] demonstrated the enormous impact of ongoing human rights abuses such as food security violations on health outcomes; for example, odds of anemia were 7.47 times higher among women from households experiencing food security violations, and a similar relationship between food security and anemia was also observed at endline. Although the difficulties of working in this setting and the relatively small population size made the measurement of more specific health outcomes problematic, future studies with better access or larger internally displaced populations might overcome this.

Finally, the evaluation of the coverage or reach of the program was based on a pre–post design, rather than utilizing a concurrent comparison group. Including a group that did not receive the services while being monitored similarly though surveys was not considered appropriate or acceptable by community partners or the implementing ethnic health leadership committees, given the known needs of all communities in this region for services. Nevertheless, the findings are still striking, especially since a secular trend in service delivery and coverage outside that which was offered by this pilot project cannot account for the substantial improvements in receipt of targeted interventions. In this setting there were no other service providers and access to the underresourced and largely nonfunctioning Burmese peripheral health system did not change during the course of the program.

A number of strengths of this project are also worth noting. The conclusions are based on data from a large number of survey respondents: over 5,000 women of reproductive age provided data over the course of the baseline and endline surveys, and over 1,500 reported specifically on pregnancies that occurred during the program period. The MOM project established a base cadre of skilled workers and community-based network upon which a broader, more comprehensive package of services might be offered in the future. Examples include adding in-home management of neonatal infections, expanding the use of misoprostol to include treatment of post partum hemorrhage, or providing services for victims of gender-based violence. Finally, the multiethnic effort provides a model for improved collaboration, capacity building, networking, and sharing of experiences, characteristics necessary for increasing both the effectiveness of public health efforts in this region [22] and increasing advocacy efforts around reproductive health rights and funding for programs.

### Conclusion

While this specific pilot project and evaluation were conducted in eastern Burma, these data demonstrate a possible model for delivering maternal and reproductive health services in other highly constrained settings. This is an area particularly lacking in documentation of innovative approaches to service delivery, not only for internally displaced communities, but more generally for low-resource settings where the international community's focus on facility-based delivery of emergency services will do little in the short term to meet the needs of a substantial subset of the population. Innovative alternatives such as the MOM pilot project are urgently needed in a wide array of settings; such approaches may maximize coverage by focusing on bringing services directly to a population in need and through expansion of the set of interventions that can be delivered outside facility settings, including components of emergency obstetric care.

## Abbreviations

ANC: antenatal care
CI: confidence interval
HW: health worker
MHW: maternal health worker
MOM: Mobile Obstetric Medics
MUAC: mid-upper arm circumference
PRR: prevalence rate ratio
SPDC: State Peace and Development Council
TBA: traditional birth attendant
